# Supplementary material for: miRTil: An Extensive Repository for Nile Tilapia microRNA Next Generation Sequencing Data
Source: Cells. 2020 Jul 22;9(8):1752. doi: 10.3390/cells9081752 (PMC7465656; doi:10.3390/cells9081752)

Supplementary Figure S1. Distribution of TargetScan Context+ scores of predicted miRNA interactions. Context+ Scores of miRNAs interactions from Nile Tilapia were obtained by TargetScan v6.0 using data from Ensembl database v.79 and miRNAs identified in Pinhal and Bovolenta *et al*. (Pinhal and Bovolenta *et al*., 2018).

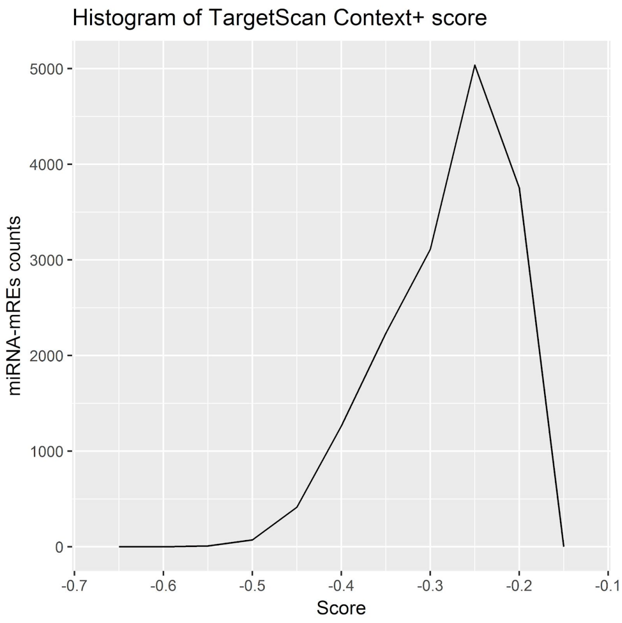

Supplementary. Figure S2. Degree distribution of nodes from predicted Nile tilapia miRNA-mRNA interactions network. The degree distribution is the probability distribution of the number of connections nodes has to other nodes over the whole network.

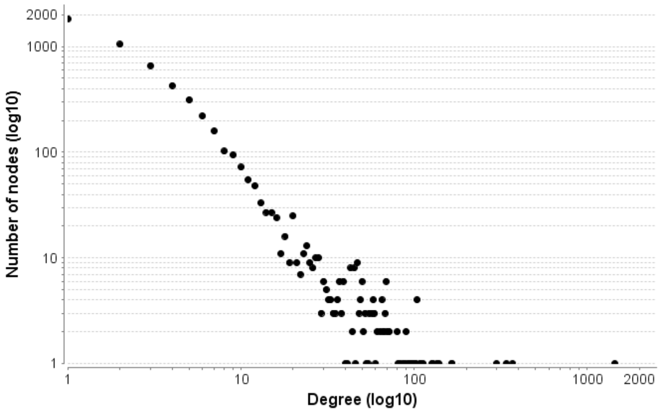

*Supplementary Figure S3. Example of the genomic location search mechanism in advanced search page***.** Users could retrieve miRNA genes using genomic window through correspondent scaffold or LG from Nile Tilapia genome and the range of start-end coordinates.

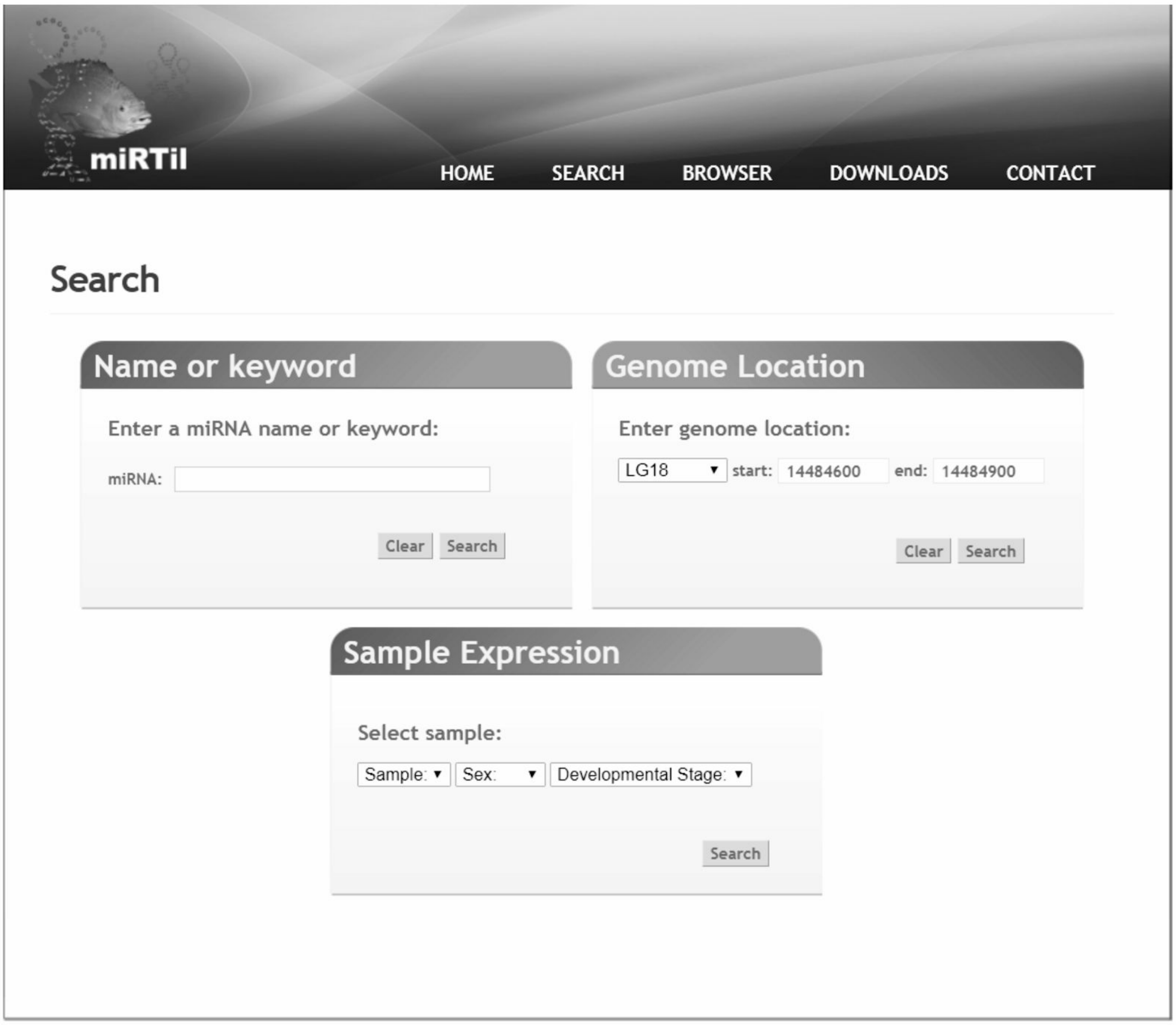

Supplementary Figure S4. Simple miRNA information resulted from the Genome location search mechanism. Page containing summarized miRNA information, such as miRNA ID, symbol and mature sequence as well as details on the genomic location, in this example to oni-mir-133a-1 and oni-mir-1-1.

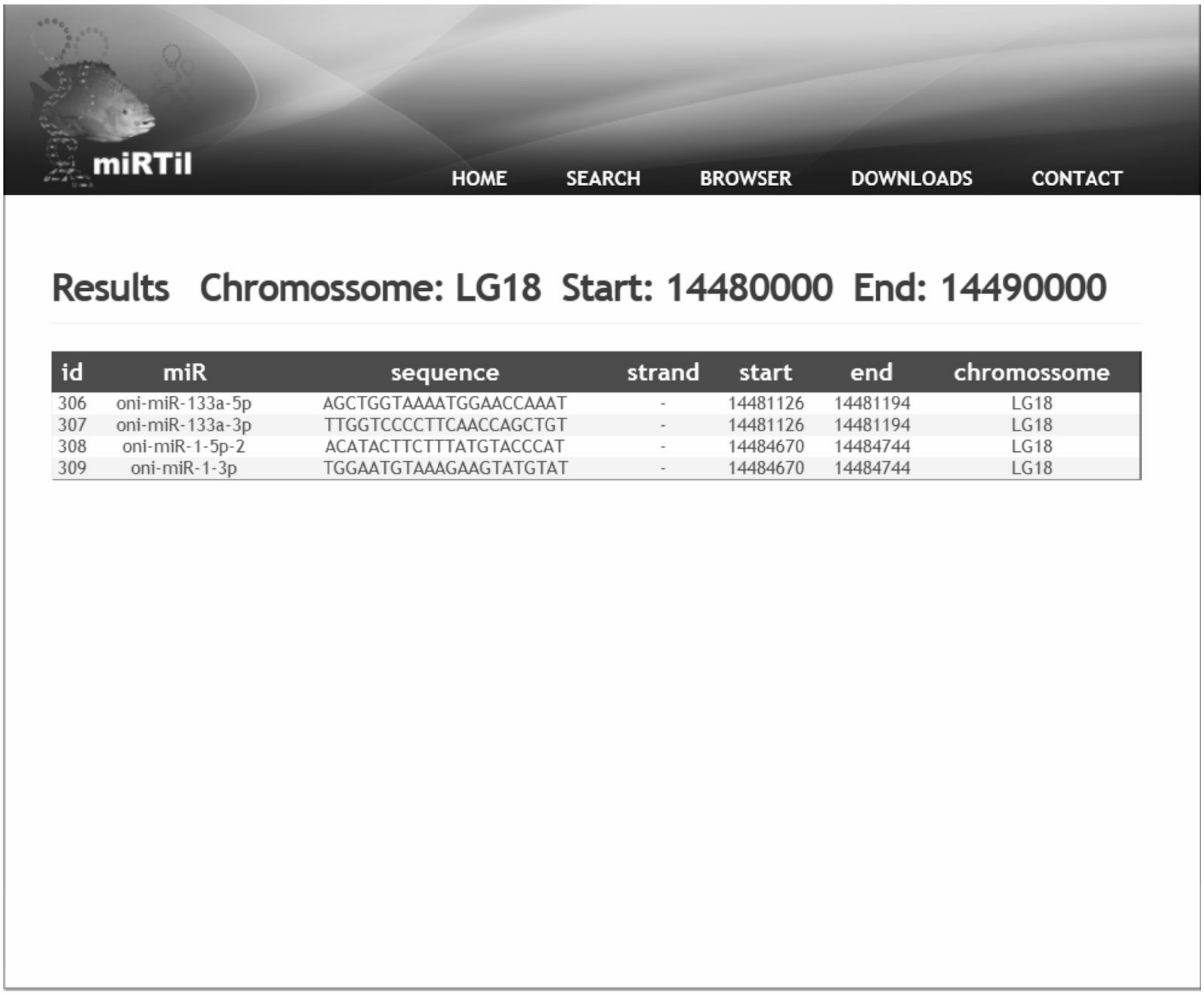

Results Chromossome: LG18 Start: 14480000 End: 14490000

id	miR	sequence	strand	start	end	chromossome
306	oni-miR-133a-5p	AGCTGGTAAAATGGAACCAAAT	-	14481126	14481194	LG18
307	oni-miR-133a-3p	TTGGTCCCCTTCAACCAGCTGT	-	14481126	14481194	LG18
308	oni-miR-1-5p-2	ACATACTTCTTTATGTACCCAT	-	14484670	14484744	LG18
309	oni-miR-1-3p	TGGAATGTAAAGAAGTATGTAT	-	14484670	14484744	LG18

Supplementary Figure S5. Example of sample search mechanism on the advanced search page. Users can retrieve miRNA genes expressed in 16 samples selecting the tissue or developmental stage, gender (male, female or sex mixed, described as "pool") and age of individuals.

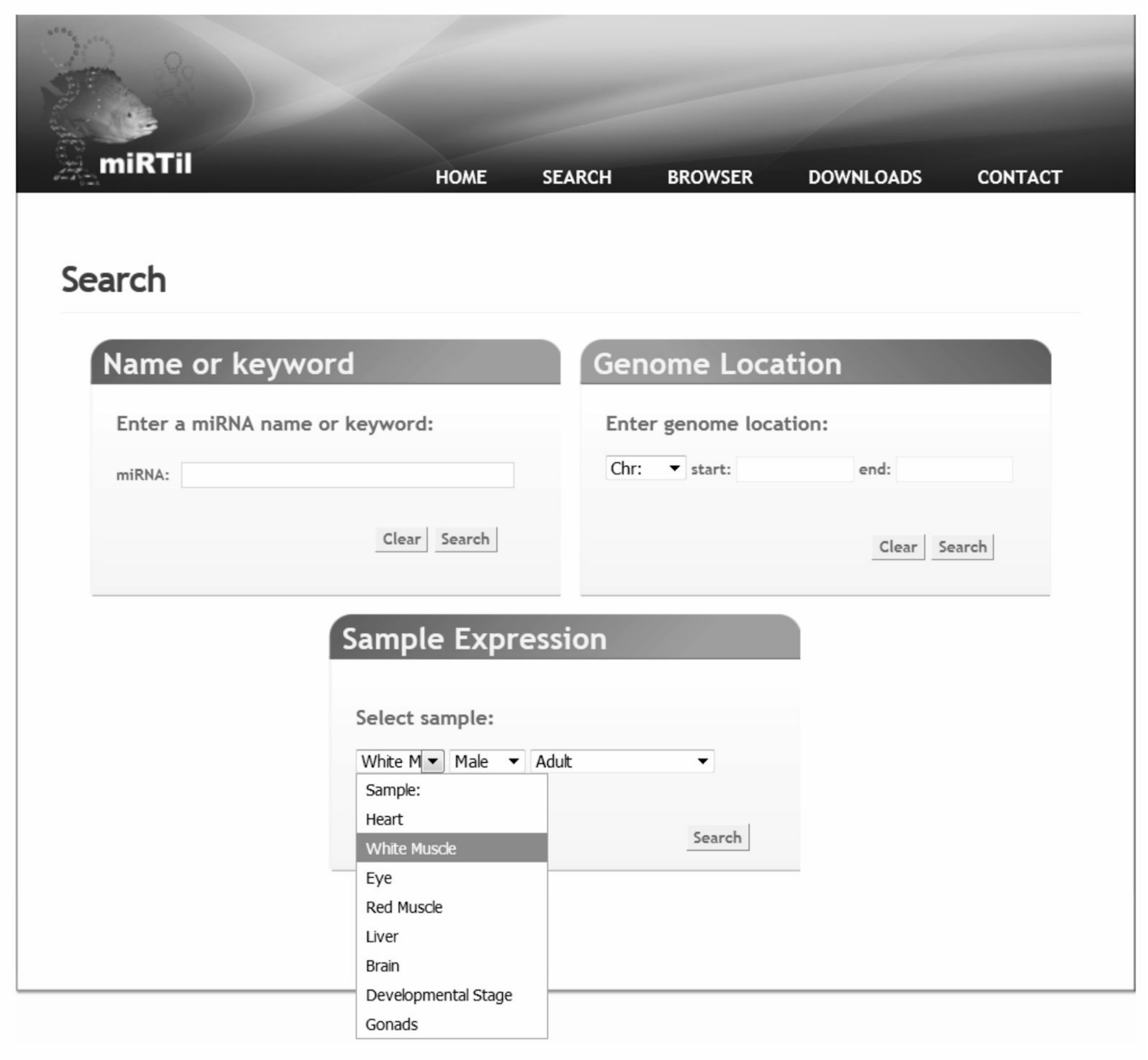

Supplementary Figure S6. miRNA information obtained by sample search mechanism. Page containing summarized miRNA information, such as miRNA ID, symbol and mature sequence as well as genomic location, in this example are partially listed 42 miRNAs expressed on male white skeletal muscle of Nile tilapia adults.

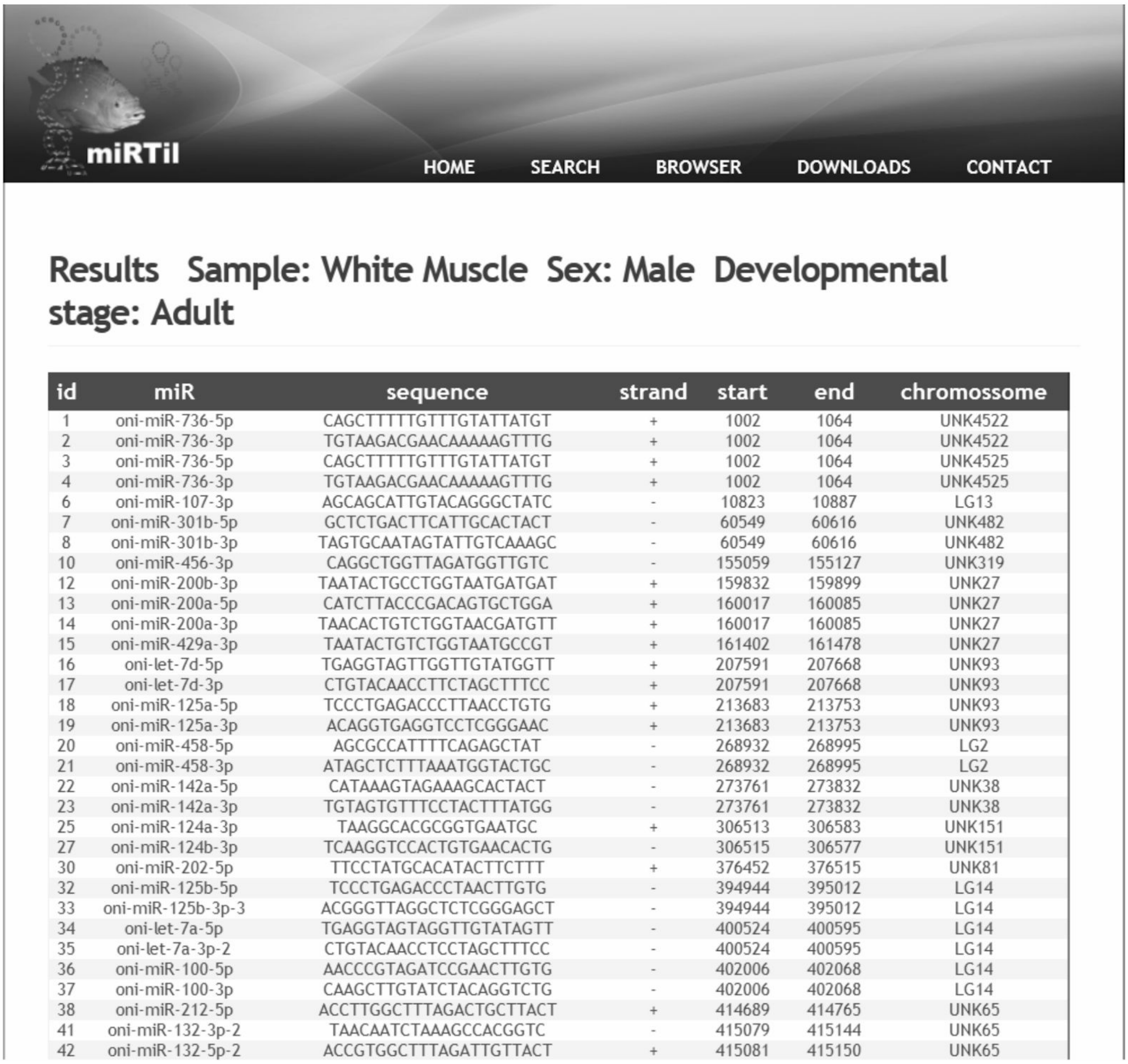

Results Sample: White Muscle Sex: Male Developmental stage: Adult

id	miR	sequence	strand	start	end	chromossome
1	oni-miR-736-5p	CAGCTTTTTGTTTGTATTATGT	+	1002	1064	UNK4522
2	oni-miR-736-3p	TGTAAGACGAACAAAAAGTTTG	+	1002	1064	UNK4522
3	oni-miR-736-5p	CAGCTTTTTGTTTGTATTATGT	+	1002	1064	UNK4525
4	oni-miR-736-3p	TGTAAGACGAACAAAAAGTTTG	+	1002	1064	UNK4525
6	oni-miR-107-3p	AGCAGCATTGTACAGGGCTATC	-	10823	10887	LG13
7	oni-miR-301b-5p	GCTCTGACTTCATTGCACTACT	-	60549	60616	UNK482
8	oni-miR-301b-3p	TAGTGCAATAGTATTGTCAAAGC	-	60549	60616	UNK482
10	oni-miR-456-3p	CAGGCTGGTTAGATGGTTGTC	-	155059	155127	UNK319
12	oni-miR-200b-3p	TAATACTGCCTGGTAATGATGAT	+	159832	159899	UNK27
13	oni-miR-200a-5p	CATCTTACCCGACAGTGCTGGA	+	160017	160085	UNK27
14	oni-miR-200a-3p	TAACACTGTCTGGTAACGATGTT	+	160017	160085	UNK27
15	oni-miR-429a-3p	TAATACTGTCTGGTAATGCCGT	+	161402	161478	UNK27
16	oni-let-7d-5p	TGAGGTAGTTGGTTGTATGGTT	+	207591	207668	UNK93
17	oni-let-7d-3p	CTGTACAACCTTCTAGCTTTCC	+	207591	207668	UNK93
18	oni-miR-125a-5p	TCCCTGAGACCCTTAACCTGTG	+	213683	213753	UNK93
19	oni-miR-125a-3p	ACAGGTGAGGTCCTCGGGAAC	+	213683	213753	UNK93
20	oni-miR-458-5p	AGCGCCATTTTCAGAGCTAT	-	268932	268995	LG2
21	oni-miR-458-3p	ATAGCTCTTTAAATGGTACTGC	-	268932	268995	LG2
22	oni-miR-142a-5p	CATAAAGTAGAAAGCACTACT	-	273761	273832	UNK38
23	oni-miR-142a-3p	TGTAGTGTTTCCTACTTTATGG	-	273761	273832	UNK38
25	oni-miR-124a-3p	TAAGGCACGCGGTGAATGC	+	306513	306583	UNK151
27	oni-miR-124b-3p	TCAAGGTCCACTGTGAACACTG	-	306515	306577	UNK151
30	oni-miR-202-5p	TTCCTATGCACATACTTCTTT	+	376452	376515	UNK81
32	oni-miR-125b-5p	TCCCTGAGACCCTAACTTGTG	-	394944	395012	LG14
33	oni-miR-125b-3p-3	ACGGGTTAGGCTCTCGGGAGCT	-	394944	395012	LG14
34	oni-let-7a-5p	TGAGGTAGTAGGTTGTATAGTT	-	400524	400595	LG14
35	oni-let-7a-3p-2	CTGTACAACCTCCTAGCTTTCC	-	400524	400595	LG14
36	oni-miR-100-5p	AACCCGTAGATCCGAACTTGTG	-	402006	402068	LG14
37	oni-miR-100-3p	CAAGCTTGTATCTACAGGTCTG	-	402006	402068	LG14
38	oni-miR-212-5p	ACCTTGGCTTTAGACTGCTTACT	+	414689	414765	UNK65
41	oni-miR-132-3p-2	TAACAATCTAAAGCCACGGTC	-	415079	415144	UNK65
42	oni-miR-132-5p-2	ACCGTGGCTTTAGATTGTTACT	+	415081	415150	UNK65

Supplementary Figure S7. Case study of miRNA with multiples samples or predicted targets detected. (A) Experimental details containing several miRNA expression evidence in multiple samples; (B) Regulatory details showing multiple computational predictions of miRNA-mRNA interactions using TargetScan v6.0.

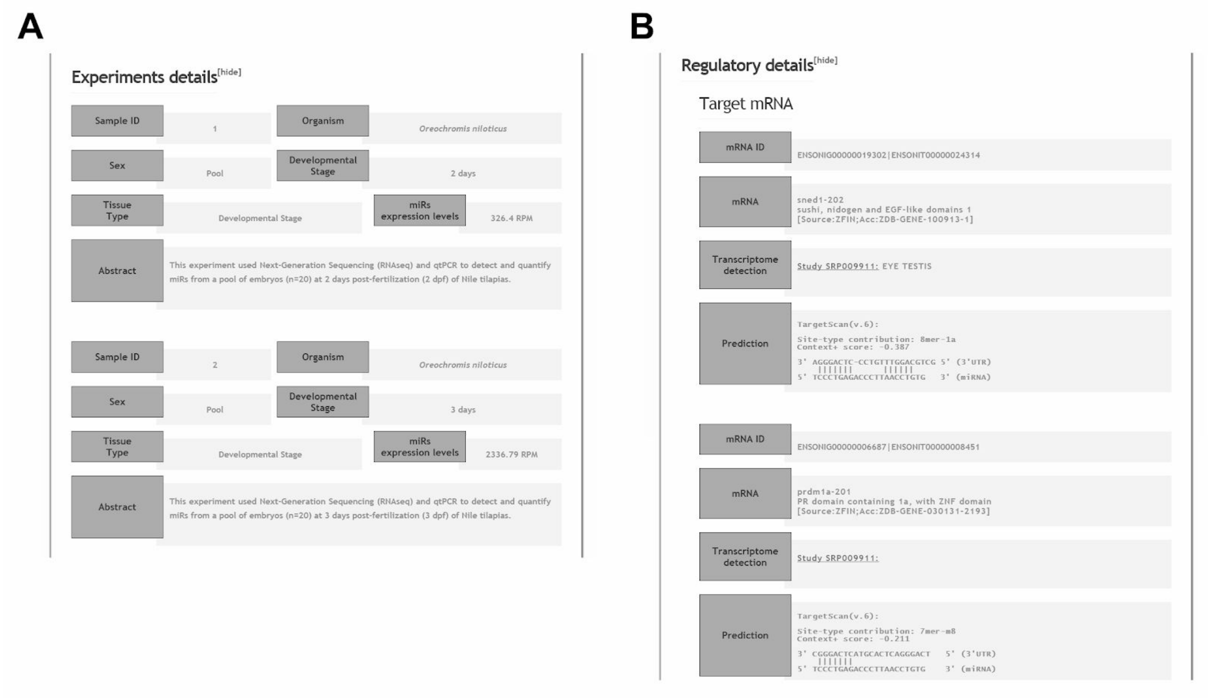

Supplement: Supplementary file 1 [file cells-09-01752-s001.pdf]
